# Health promoting settings in primary health care - "*hälsotorg*": an implementation analysis

**DOI:** 10.1186/1471-2458-10-707

**Published:** 2010-11-17

**Authors:** Amina Jama Mahmud, Ewy Olander, Lovisa Wallenberg, Bo JA Haglund

**Affiliations:** 1 School of Health Science, Blekinge Institute of Technology, SE- 371 79, Karlskrona, Sweden; 2 Department of Public Health Sciences, Karolinska Institutet, SE-171 76, Stockholm Sweden

## Abstract

**Background:**

Sweden, like many other western countries, faces increasing rates of lifestyle related diseases and corresponding rise in costs for health care. To meet these challenges, a number of efforts have been introduced at different societal levels. One such effort is "*Hälsotorg*" (HS). HS is a new health promotion setting that emerged in collaboration between the Swedish County Councils and Apoteket AB, a state-owned pharmacy company. HS's overall aim was to improve population health and facilitate inhabitants' responsibility for self-care. A new National Public Health Policy, introduced in 2008, emphasizes more focus on individual's needs and responsibility as well as strong need for county councils to provide supportive environment for individual-centred health services and increased health literacy among the population. In light of this policy, there is a need to examine existing settings that can provide supportive environment for individuals at community level. The aim of this study was to explore HS's policy implementation at local level and analyse HS's activities, in order to provide a deeper understanding of HS's potential as a health promoting setting.

**Methods:**

Materials included a survey and key documents related to the development and nature of HS on local and national levels. A policy analysis inspired by Walt and Gilson was used in data analysis. In addition, an analysis using the principles of health promotion in relation to HS policy process and activities was also carried out.

**Results:**

The analysis illuminated strengths and weaknesses in the policy process, its actors, contextual factors and activities. The health communication approach in the analysed documents contained health promoting intentions but the health promoting approach corresponding to a health promoting setting was neither apparent nor shared among the stakeholders. This influenced the interpretation and implementation of HS negatively.

**Conclusions:**

The analysis indicates that HS has potential to be a valuable health promotion setting for both population and individuals, given the strong intentions for a health and empowerment building approach that is expressed in the documents. However, for a more sustainable implementation of HS, there is need for an in- depth understanding of the health promotion approach among HS stakeholders.

## Background

Sweden, like many other western countries, faces increasing rates of lifestyle related diseases and corresponding rise in costs for health care. In order to meet this challenge, a number of efforts have been introduced at different societal levels. At the national level, the government introduced a new national public health policy [1] in which other actors such as the Pharmacy (Apoteket AB) were invited to take a more active role in health promotion and disease prevention. It was in line with this, that a new health promotion setting or a healthy living centre named *Hälsotorg *(Health Square (HS) in English) emerged in collaboration between "Apoteket AB", a state-owned pharmaceutical company and county councils as a new setting based Primary Health Care (PHC) activity.

A new National Public Health Policy bill [2] introduced by the centre-right coalition government in 2007, marked a transition from strong focus on social structures and health determinants to more attention on individuals [3]. The overall aim of the new bill was to create social conditions to ensure good health on equal terms for the entire population, as stated in the former public health policy (Govern Bill 2002/03:35) however, the new policy focuses more on health behaviours and individual's responsibility for health [3]. The new policy also emphasizes stronger local health promotion initiatives in different settings, one of which is a more health promoting oriented health services. Recently the Swedish Government also introduced a new Health and Medical Care Policy [4] with objectives to offer accessible and effective health care based on individuals' needs, and transferring the choice of care provider to the individuals. These policies force county councils and regions to provide a supportive environment for individuals to make informed health related choices and to provide available efforts for individual-centred health services. The utmost goal is to increase health literacy skills among the population. The question we are asking in this paper is if the new Swedish HS could be such a health promoting initiative in Primary Health Care (PHC) that could meet and support both community and individuals at different levels of health literacy [5].

HS is a meeting place for universal health information and individual health counselling on life style related issues with health professionals, health measurements, group activities, and access to trustworthy internet based health information sites and lifestyle tests. HS also provides guidance to appropriate care providers. The origin of HS can be traced internationally to the "community pharmacies movement", i.e. re-orientation of pharmacies into a health promoting service [6] and to "Healthy City shops"; a Danish Healthy Cities Network initiative for health information and guidance in health issues [7]. Comparison could also be drawn with "Healthy Living Centre's" in the United Kingdom [8]. These meeting places with community health service and health programs aimed to promote health in its broadest sense, target disadvantaged groups and tackle inequalities in health.

Settings for health is defined as "the place for social context in which people engage in daily activities in which environmental organizational and personal factors interact to affect health and wellbeing"[[9]:pg.19]. A settings approach provides an ecological perspective where local context networking and alliance building is important [10]. This approach is built upon the principles of health promotion, of empowerment, participation, holistic, intersectional, equity, sustainability and multi-strategy [11,12], and health promotion as a process for enabling people to increase control over, and to improve their health [9].

Settings approach has gained importance in the past decade and has enjoyed sustained support in international health promotion research and discussions [13,14]. Similarly local evaluations of HS, have considered it as a valuable setting for health promotion and a complement to traditional health and medical services [15-17]. HS, according to these local studies, is well accepted as a strong health promotion effort among local communities and HS personnel. However, evaluation of settings based health promotion is not an easy task as settings are complex contexts with many actors whose varied interests and expectations can influence the implementation process [18]. There is therefore a need to understand the implementation process of HS to corroborate or contradict the results of the local evaluations before further expansions is considered. In the light of the Swedish governments' request for more local health promotion initiatives and a more health promoting health services there is need for further studies of HS and HS's potential as a supportive environment that could strengthen health and health literacy in the community [2,19].

The aim of this study was to explore the HS policy implementation on local level and to analyse HS's activities, in order to provide a more in depth understanding of the HS's potential as a health promoting setting.

## Methods

The study adopted a multi-method design [20]. Materials included a survey to HS personnel, and key documents on national and local levels related to the development and character of HS. A policy analysis triangle framework inspired by Walt and Gilson [21] was used for the data analysis. In addition, an analysis using the principles of health promotion in relation to the activities of HS was carried out.

An electronic survey was distributed to key persons at 30 HSs in Sweden in 2006. A pilot test of the questionnaire was conducted prior to the survey. Twenty-one HSs responded initially and five more responded after two reminders. In total, 26 HS responded representing 13 of 18 county councils and two regions. Four HS did not respond, two claimed lack of activities, and two cited lack of time. The questionnaire consisted of 25 closed questions with opportunity to comment on each question, and four open-ended questions focusing on; HS's intentions and objectives, collaboration and agreements, personnel and activities and development and evaluation.

Key documents encompassed national and local policy documents, meeting protocols, an Apoteket AB Action plan and an Apoteket AB internal report related to the development of HS. Current national key policy documents on national levels encompassed the National Public Health Strategy for Sweden - A Green Paper [22]. The local policy documents included a total of 11 local policy documents; two regional/county council policy plans for 2006, one local primary health care policy plan for 2005, four municipality plans including local public health plans formulated in 2005 and four local HSs' contracts formulated between 2003-2006. The documents were obtained on request in the survey and by internet search with the search terms "*Hälsotorg" *and "Apoteket" (Pharmacy) from the involved county councils and municipalities' web pages. The local documents represented ten HSs in four regions/county councils.

Data from the survey, documents and internet searches were analyzed with quantitative and qualitative content analysis [19,23]. Thereafter Walt and Gilson's "policy analysis triangle framework" [21] was used to analyse components in the policy triangle, the policy making process (problem identification, issue recognition, policy formulation, policy implementation, policy evaluation), actors, content and contextual factors, and its complex interaction.

## Results

### Problem identification and issue recognition

Policy documents and survey response indicated that the implementation of HS arose from a need for structural changes due to high patient load on health services and request for enhanced health services, especially the PHC. Two main rationales for implementation of HS in Sweden were pointed out. These were; the government's responsibility for public health as indicated in national policy goals, and the uncertainty in health services' ability to overcome health challenges i.e. escalating costs of prescribed drugs, lifestyle related health problems, health differences within the population and increasing rates of sick leave. The national public health policy documents and the Apoteket AB Action plan identified health information as a vital area that needed improvement in order to increase accessibility to health communication and raise health literacy among the population.

### Policy formulation and implementation

A National Public Health Strategy for Sweden - "a Green Paper" [22] published in 2000 proposed Public Health Goals addressing structural and environmental health determinants. One objective focused on a more health promoting Health and Medical Services and their key functions in health promotion. Another focused on access to accurate health information as a prerequisite for equitable good health. Apoteket AB was pointed out as a public health actor with the responsibility to provide producer independent, accurate and accessible health information to pharmacy customers.

These statements marked a starting point for Apoteket AB's "Health Dialogue" (HD) projects intending to involve pharmacies actively in public health work and to contribute to improved long-term public health. The HD experiences laid foundations for formulation of an Apoteket AB Action plan 2002 and the HS concept. The Action Plan declared HS as a *s*etting for health in which partners (local pharmacies, primary health care and municipalities) could allocate resources and enable long- term health dialogue with the population. Furthermore, the Action plan indicated that a HS should be located near a pharmacy, or as part of ordinary pharmacies, in primary health or municipality centres to profit from the pharmacy customer flow.

The former Swedish Public Health Objective Bill [1] was the third important national policy document for the HS development. The bill diminished Apoteket AB's responsibility for health information and stated that responsibility for health information should rest on all relevant public sectors allocated with this responsibility. The policy also stated that all sectors of society should bear responsibility for supplying of health information at no cost to the population and should support healthier living. The Pharmacies were however, encouraged to continue taking advantage of the big flow of customers visiting them to provide objective and accessible health information.

According to the Apoteket AB Action plan, 30 HSs were established between 1990-2005 in 18 of the 21 county councils in Sweden. Two HSs started during the late 90's as pharmacies with expanded health information activity using district nurses as a health counselling resource. Most of the responded 26 HSs were established between 2003 and 2005 (table 1). An additional 25 were planned. The ambition was to establish one large HS in each county council nationally.

**Table 1:** HSs included in this study and the year of establishment

Year	1990's	2001	2002	2003	2004	2005	Total
**Number of **HS	2	1	3	5	8	7	26

At the time of this study, seventeen HSs were integrated into the ordinary day to day activities of the PHC or the Pharmacy (depending on the location), while one HS was in project form. Respondents at eight HSs were uncertain if their HS was in project form or an integrated ordinary activity. At local level, policy formulation emerged in discussions between local pharmacies and county councils but, this process was mainly guided by the Apoteket AB Action plan. Contracts with Apoteket AB were finalized mostly at local level. It is not clear as to what extent HSs' personnel, interest groups; patient or community associations were involved in the local policy formulation. Thirteen of the responding 26 HSs had written agreements with county councils, 10 had verbal agreements and three had none. Four HSs also had additional agreements with municipalities and one with private companies.

The common overall aims for HS in the investigated local policy plans were to improve population health, facilitate inhabitants' responsibility for self-care, and to be a meeting place for health promotion. Survey respondents considered that local aims and activities corresponded well with those formulated at the national level. Five categories of objectives for HS emerged in the local documents: *partnership, personnel, finance, activity *and *community health promotion*. The partnership category focused on improvement of intersectoral collaboration and creation of shared settings for public health actions mainly between the pharmacy, county councils and municipalities. Other actors could be invited to participate in specific HS's activities and in production of health information material. The personnel category focused on HS's agreements including participating personnel professions, stipulated time for duty at HS and education for HSs' personnel. The finance category focused on partners' responsibility for costs such as salaries, offices, computers, health information material, tests and evaluations. The activity category focused on mediating accurate and contemporary health information, supporting self-care and strengthening individual actions for health with an emphasis to healthy eating habits, physical activity, use of tobacco and alcohol and promoting sexual health, in order to correspond to the national health goal objectives. Finally, the community health promotion category focused on commitment to use HS as a setting for community actions that could support healthy lifestyles for individuals, groups and communities.

The concept of "health promotion" was used positively to express a new approach for a more health- promoting health and pharmacy service throughout the documents and survey response. Different expressions such as "*promoting health"*, "*risk and disease prevention" *and "*public health work" *were used to express HS's activities. Also, survey respondents used different terms to explain HS objectives, activities and implementation. Survey respondents expressed the need for clear guidelines as far as HS's activities were concerned. According to the respondents, there was a gap between the stated objectives in the policy documents and how they were implemented in the practice i.e. HSs. Common reflections on health promotion resulted in comments like, "*unclear objectives could weaken a common approach*" and "*inability to act according to national aims*".

The implementation process could be seen as both a top down as well as bottom up process. Top down in the sense that the national Apoteket AB governed the HS's structure and content. Bottom up, as the HSs' groups were established with representatives from local pharmacies and county councils including HSs' personnel.

### Content

The HS's activities were based on a "basic package" stipulated in the Apoteket AB Action plan and objectives in local agreements. Most HSs offered health information in form of pamphlets, access to a customer "health computer" with quality assured health information and tests, and individual health counselling free of charge. Almost all HSs provided health products for purchase, equipment for visitors to measure weight, BMI, waist and blood pressure, and health profiling with coaching at a cost. Several HSs had specific topic activities usually organised in recurrent announced "theme weeks" with local representatives from governmental and non-governmental organisations (NGO's). Depending on local available resources, service for physical activity prescriptions, lectures or study circles, organised physical activities and Nordic walking were offered. Only a few respondents discussed a more focused activity for target groups and chronically ill in accordance with the objectives in the national Apoteket AB's documents. Most respondents pointed out easy access to objective health information, and to individual health support of community inhabitants to increase personal responsibility for health, as the most prioritized of HS's activities.

### Stakeholder and Actors

The analyses of documents and survey response indicate that a number of actors were involved at different levels in the HS's policymaking process, referred to as "stakeholders", "partners", "interest groups" and "active HS's actors". Both the central pharmacy chain and personnel at local pharmacies were involved. County councils and primary health services were represented by managers and personnel at HS. These include nurses, physiotherapists, dental hygienists, occupational therapists. The municipalities' health and social sector were mainly represented by health planners.

At local level, county councils and public primary health services were important key stakeholders as they had the responsibility for implementing HS's activities into practice in order to fulfil national and county council aims and objectives of " a more accessible and health promoting health services" [1]. Apoteket AB appeared as the most powerful "policy keeper" and "agenda setter" for the establishment of HSs and the activities. The central Apoteket AB manager took part in the establishment of all local HSs and the central Apoteket AB and pharmacy personnel were constantly present actors at local HSs. In this way, Apoteket AB kept power and took immense responsibility.

The Swedish Government, the Swedish National Institute of Public Health, Swedish Municipalities and County Councils' Association were mentioned as stakeholders with interest in the HS's development, in both the Apoteket AB Action Plan and by the survey respondents, but could not be identified as "active actors" in the policy formulation or implementation. Neither were governmental organisations nor NGOs, local municipalities and inhabitants mentioned as partners in the documents. According to the survey respondents "the active HSs' actors" on local level, mainly consisted of pharmacy and county council personnel and mostly district nurses. Their presence varied from daily to one day a week, depending on the collaboration agreement. The local documents indicated that county council personnel were more active in the HSs situated at primary health centres. This was also corroborated by the survey respondents who confirmed that HSs located in PHC centres involved other health professionals such as dental hygienists, occupational therapists and physiotherapists in their activities from time to time. Due to differences in involvement, county council actors' power and responsibility varied in relation to the pharmacy actors from one HS to another. Non-governmental organisations were or could be invited to participate in different theme-weeks and campaigns.

### Policy evaluation

Neither policy documents nor survey respondents provided a clear indication on how the implementation of HSs should be or were monitored and evaluated. According to the survey respondents, most HSs had follow-up meetings at least once a month to reflect on progress and planning although local policy documents and contracts made little provision for this. Four HSs reported comprehensive annual evaluations, while three had occasionally conducted a comprehensive evaluation. The Apoteket AB internal report therefore, requested coordinated evaluations for comparisons and more in depth evaluations regarding evidence and cost-effectiveness of HS's activities.

### Contextual factors

Several contextual, structural, situational and exogenous factors affected the implementation of the HSs. The "Green paper" [22] and the Swedish Public Health objectives [1] had particular influence on the development of HSs as they highlighted lifestyle determinants, the need of improvement of health information, and a more health promoting health services. The "Green paper" as well as the Apoteket AB Action plan also referred to an international discourse for more health promoting health services and pharmacies. These discourses supported to a large extent the HS's policy process and gave it high legitimacy.

Another contextual factor was the shared responsibility for HSs between two principal founders; Apoteket AB and county councils. Apoteket AB had a central steered management while the county council had a local steered management. Apoteket AB also had a dual role: to sell pharmaceutical products as a profit-making concern and at the same time, provide health information to reduce consumption of prescription drugs. These overt differences and Apoteket's seemingly incompatible dual role gave at times conflicting roles and could affect the HSs' activity and decisions, according to the survey respondents. However, this was not considered as problematic. Survey respondents also narrated that HS progress and transition after the project phase was affected by lack of adequate resources and guidelines which resulted in little time for planning, development and evaluations.

The populations' need for HSs' services, availability of qualified personnel and the possibility to host activities affected the resource allocation and establishment of HSs. A political will and clearly stated collaboration plans in the local policy plan, acted as catalysts for establishment of new HSs. Furthermore, presence of municipality health planners seemed to be a supporting factor for municipality participation in HSs' establishment. All four collaborating municipalities who had HS's agreements with Apoteket AB and county councils had a health planner, responsible for the municipality public health work, in their workforce.

## Discussion

The documents and the survey express ambitions to strengthen local health promotion activities in line with the Swedish Public Health Policy objectives [3]. The survey respondents pointed out health benefits with HS activities and see HS as a vital physical location for health promotion activities. The rapid and extensive diffusion of HSs in 18 of 21 regions/county councils, indicates a strong local political will for implementation of HS as a valuable investment for health promotion efforts. However, the local policy documents were short of origins for health promotion and connection to national and international health policy goals and objectives. In this way the underpinning meaning of a health promoting settings approach [24] does not seem obvious to the HS stakeholders and actors. This could diminish the understanding of Hs' health promotion roots as it is important that fundamental philosophical values of health promotion are clearly reflected in the structures that create a supportive environment for health promotion [25] The health promotion principles [11], were more or less explicitly mentioned and reflected in documents and by the survey, respondents but not consciously discussed as foundations for the HSs. Such indistinguishable foundations could reduce the possibilities for a consistent and shared health promoting settings based approach among HS's stakeholders and actors [26].

In the following discussion the health promotion principles; intersectoral collaboration, participatory, empowerment, holistic multi-strategy, equity and sustainability; will be used as a base for reflections of HS's potential as a health promotion setting.

### Intersectoral collaboration

Although collaboration between different actors and organisations was highly pursued, collaboration was mostly established between Apoteket AB and county councils. Municipalities and NGOs were recognised as important HS actors, but were hardly involved in the policy making process. Apoteket AB was strongly driving their own agenda which could give the impression that HS is an Apoteket AB- project and not a collaborative project, especially when HSs were located in pharmacies. This caused a power imbalance between the different stakeholders. This power as a "thought control" [21] could diminish local authorities and organisations feelings of participation and responsibility for implementation and sustainable development of HSs in ordinary Health Services. Also, an unclear financial cooperation, various employment and financial situations, values and interests made the collaboration into a balancing power act between Apoteket AB, county councils and municipalities. To avoid conflicting interests, there is a need to hold on to agreements and underpinning HS values of health promotion and settings approach [12,13]. As these agreements are vague and inaccessible to all actors in the case of the studied HS, must management of such barriers be the main task for the collaborating partners as the people working in the practice or the "implementers", will need a lot of management support [27].

### Participation

In both the documents and the survey, stakeholders and actors participation were emphasised as a strong concern for the implementation process and collaboration in HS's activities. The survey respondents considered their work to be individual-centred, with a high level of participation whereby HSs' visitors had influence on the consultation mode and right to take part in decisions concerning their own health. In reality the municipality representatives, NGO's and HSs' visitors mostly participated as invited by Apoteket AB and county councils on their premises. This gave them a lower participation level than the pharmacy and county councils actors. Such limited involvement could weaken the health promotion settings approach, where activities are supposed to be guided by local needs and context expressed by the people whom these interventions are meant for [28]. More involvement of stakeholders, HSs' actors and community actors could strengthen an understanding and adaptation of HSs, and development of HSs to a more comprehensive settings based approach [29].

### Empowerment

The survey respondents considered that the HS's efforts with easy access to health information, manned HSs and individual consultations with a participatory individual-centred approach shaped opportunities for empowerment processes and support of individuals' capacity to take responsibility for their own health. These efforts are in line with the local HSs' policies and the national public health [3] which stresses empowerment and support as important components for public health improvements [30]. Support of such empowerment processes for increased control over decisions and actions for health [31] requires a professional empowerment approach, based on the beliefs of its philosophic assumptions and skills to use them as foundations for health communication [32].

The vague awareness of health promotion concepts that emerged in the analysis could weaken the foundations for a professional empowerment approach that is essential for improvement of health literacy. These include; personal, cognitive and social skills, and the ability to get access to, understand and use information for promoting and maintaining health [19,33]. Nutbeam [18] points out health communication as essential in modern health promotion and the improvement of health literacy in a population. The need for supportive environment like the HS, will probably increase in the future as demands on people to take more responsibility for their personal health increases [2]

### Multi-strategy

The HS's strategy with a variety of activities and approaches corresponds well with a narrow interpretation of the health promotion "multi strategy" principle [12]. The organisational change to a HS with intentions to increase community collaboration, several activities, population and individual approaches, health information and health counselling, and promotion and prevention, gave a wide range of efforts. This requires a committed and composed health promotion approach among HS's managers and personnel to be a health promoting setting [28]. Health promotion concepts were not used in a consistent mode, which implicates a diffuse or insufficient understanding of the concepts, its origins, values and meaning. This affects HSs' actors' interpretation of approaches, roles, efforts and activities, and chances for a joint approach.

Health communication is a vital part of the HSs' activities and their visitors have a wide choice of health information materials, opportunities to discuss the information with HS's personnel and possibility for scheduled individual health counselling. HSs' personnel work with both "population based, expert led health communication" and "individual based health communication focusing on individual's needs, health literacy and request of support". This double mission stresses the need for awareness of differences in approaches and how to synthesise them in the HS meetings [34]. Lack of awareness of the different approaches among HS personnel could result in a dominant expert lead "individual health advice giving" -contrary to the empowerment health promotion approach [35].

### Holistic

The principle of "holistic" [11] takes account of physical, as well as mental, social and spiritual health and its interactions. Local policies and surveys show ambitions for a holistic settings based approach [36] for the HS's activities and its role as an important actor in the community health promotion context. In practice, this holistic approach was not so apparent. An example is HSs' collaboration with other local settings such as schools and workplaces were mentioned but not elaborated upon. Such collaboration between settings is important in order to achieve a holistic approach, because people belong or move between different settings [13,36]. Within HSs most described HSs' activities focused on physical health and lifestyle changes. This implicates a traditional bio-medical preventive approach, despite the expressed ambitions in the documents to work with a more holistic settings based approach. This could be attributed to the diffuse understanding of health promotion foundations, and its relation to disease prevention in practice [37].

### Equitable

In relation to the health promotion principles of equity and social justice [12] the HSs' local policies emphasise health on equal terms for entire population in conformity to the overall national public health goal [3]. Working with both a population and an individual approach put the interpretation of equity to the fore; "equal in the sense that same efforts for all and after each individual needs" [ibid]. The accessibility to HS's services and health information, free of charge for all HSs' visitors, and opportunities for individual support was seen as a way to meet this call of equitable health promotion request. Frohlich and Potvin [38] argue that intersectorial collaboration and participation are important for a vulnerable population approach In the case of HS community members or patient representatives were not involved in the implementation of HSs' and as such demonstrate that this approach is not well integrated into the implementation of HS despite the opportunity available in their collaboration.

### Sustainability

Sustainability i.e. "bringing about changes that individuals and communities can maintain once initial funding has ended" [12] was a noticeable issue for several HSs. HS' long term activities; effectiveness and sustainable efforts were discussed as a strong predictor for further development of health square activity, but not much time was used for follow-ups, evaluations and developmental work.

A weakness that could make it difficult to plan for further development of existing HSs and implementation of new ones nationwide, is the lack of standards for assessment of HS activities. A set of standards to guide the HS evaluations and quality development can be useful, in the initial stages of planning of any project [39]. Hence it is important to incorporate formative, process and outcome evaluations to shed more light on HS' practical work.

Development of policies are important for creation of supportive environments [40], the scarcity of health promoting policies amongst involved sectors weaken maintenance of newly established settings such as HS. The analysis of the contextual factors shows that different internal and external factors influenced the implementation and activities in HS. Earlier studies have also shown that contextual, internal and external factors as well as organisational capacities and conditions influence the adaption of a new approach and the implementation process [18,41,42].

### Limitations

The electronic survey questionnaire was only piloted once. Additional pilots would probably have increased the validity and reliability of the study. Furthermore, the policy and other documents attained were few compared to the number of existing HSs. Hence, the results may not reflect all varieties of HSs. However, it could be argued that since the documents represented more than half of the established HSs, the result is fairly representative. This shortcoming, nevertheless, has been taken into consideration in the analysis. The authors avoided making comparisons between county councils/regions and from drawing far reaching conclusions about how the local documents gave legitimacy to HS implementation and transformation of HSs from project to integrated activities in their respective practices.

## Conclusions

The study contributes to an understanding of the new setting HS, as well as the HS's policy processes, its actors and context, and how this could be better framed to support implementation and sustainability. The Walt and Gilson policy analysis triangle [21] was a useful tool for the policy analysis of local implementation, although the analysis model is discussed by the authors as a tool for understanding more exhaustive policy making processes. The health promotion principles used as basis for the analysis contributed to a deeper understanding of the policy implementation and practice of HS.

The analysis suggest that HS has potential to be a valuable health promotion setting as the findings show that there are keen intentions to organise HS using health promotion and empowerment building approach. However, this is not so apparent nor is it shared among the actors. Recent deregulation of Apoteket AB [43] makes sustaining or further development of HS activity in the original collaboration, uncertain. On the other hand this changed circumstances, can be seen as a window of opportunity for the PHC to boost its health promotion efforts by taking over the responsibility for HS activity. This would contribute to a more health promoting health services as stipulated in the new public health policy [2]. To facilitate this process there is a need for a better understanding of health promoting settings approach among the HS's actors, politicians as well as HS workers. Furthermore, to be able to sustain HS, there is a need to understand implementation process and the importance of carrying out systematic evaluations in the practice

## Competing interests

The authors declare that they have no competing interests.

## Authors' contributions

The individual contributions of authors to this manuscript are; LW, EO, BH, AJM conception and design; LW, EO acquired the data; LW, AJM, EO, BH analysed and interpreted the data; AJM, EO BH drafted and revised the article. All authors read and approved the final version of the manuscript.

## Pre-publication history

The pre-publication history for this paper can be accessed here:

http://www.biomedcentral.com/1471-2458/10/707/prepub
